# Research on the Hydration Mechanism and Mechanical Properties of Stainless Steel Slag–Fly Ash Recycled Concrete in Pavements

**DOI:** 10.3390/ma18214906

**Published:** 2025-10-27

**Authors:** Liuyun Huang, Sixian Chen, Zhuxin Lan, Yuliang Chen, Tun Li

**Affiliations:** 1 School of Civil Engineering and Architecture, Guangxi University of Science and Technology, Liuzhou 545006, China; 100000635@gxust.edu.cn (L.H.); ylchen@gxust.edu.cn (Y.C.); 100000644@gxust.edu.cn (T.L.); 2 China Petroleum Engineering & Construction Corporation Southwest Company, Chengdu 610041, China; 13098778576@163.com

**Keywords:** stainless steel slag (AOD slag), recycled concrete, hydration mechanism, mechanical properties

## Abstract

This study systematically investigates the effects of slag from the argon–oxygen decarburization (AOD) process, fly ash, and recycled aggregate (RA) replacement ratios on the mechanical properties of mortar samples and AOD slag–fly ash recycled concrete. The sustainable reuse of industrial by-products and construction waste is significant for reducing environmental impact and resource consumption during pavement construction. Experimental results demonstrate that when AOD slag and fly ash are used in combination, they undergo synergistic hydration reactions, producing calcium hydroxide (CH), calcium silicate hydrate (C-S-H) gel, and ettringite (AFt), resulting in superior strength compared to the individual use of either material. This research reveals that concrete strength decreases significantly when the recycled aggregate replacement ratio exceeds 50%; therefore, RA = 50% was selected as the optimal replacement ratio for subsequent studies. On this basis, when the combined replacement ratio of AOD slag and fly ash is 10–20%, concrete performance reaches its optimum level: maximum compressive strength is 33.9 MPa, which is 8.57% and 36.2% higher than using fly ash or AOD slag alone, respectively; maximum flexural strength is 4.6 MPa, which is 6.08% and 14.44% higher than using fly ash or AOD slag alone, respectively; and peak axial compressive and splitting tensile strengths are 24.9 MPa and 3.4 MPa, respectively. These findings demonstrate that the synergistic use of AOD slag, fly ash, and recycled aggregates can produce concrete that meets pavement application requirements, while effectively promoting the resource utilization of industrial by-products and construction waste, aligning with circular economy principles.

## 1. Introduction

Road infrastructure plays a crucial role in promoting economic development and improving transportation systems. Concrete, due to its high strength and durability, has become a common material in road pavements. However, the accelerated construction of urban and rural roads, shortage of natural aggregates, and high carbon emissions caused by cement production pose significant challenges. Meanwhile, the industrial and construction sectors generate large amounts of solid waste each year, such as slag from argon–oxygen decarburization (AOD) during stainless steel refining and construction and demolition waste. If not properly managed, such waste leads to resource misuse and land occupation while causing environmental risks such as dust pollution and heavy metal leaching [1,2,3]. Therefore, utilizing waste as construction material is a feasible approach that balances ecological and economic benefits.

Research indicates that grinding stainless steel slag into powder and using it as a cementitious material is feasible. Some scholars [4,5] have studied the feasibility and safety of using stainless steel slag as a cement admixture. However, these studies focused primarily on general material properties and safety considerations, without specifically addressing pavement applications. The potential synergistic effects of using stainless steel slag and fly ash in recycled concrete for pavement construction remain largely unexplored. This research is critical for developing sustainable and cost-effective pavement materials. Stainless steel smelting slag, with β-dicalcium silicate (β-C_2_S) and magnesia-silicate calcium stone as the main components, has been proven to have cementitious activity and can be used to prepare composite cement that meets the requirements of road engineering standards. Ana C P et al. [6,7] evaluated the potential of stainless steel AOD slag as a supplementary cement material, which cannot only recycle solid waste but also reduce carbon dioxide emissions in cement production. Dai Jian et al. [8,9] found that the compressive strength of cement mortar experiences a continuous downward trend with the increase in AOD slag content, and determined its optimal substitution ratio, identifying weak cementitious activity in AOD slag. Thus, resource utilization efficiency needs to be improved through activity activation methods. Luo Zhongqiu et al. [10,11,12] fabricated cementitious materials by compounding stainless steel slag, finding that it could interact and activate fly ash. Microscopic observations revealed that the products provide the materials with excellent mechanical properties. Moreover, through a dual mechanism of chemical adsorption and physical encapsulation, the leaching concentration of chromium ions was significantly reduced, complying with relevant standards and specifications [13]. This provides a basis for the resource utilization and safe treatment of stainless steel slag. In conclusion, stainless steel slag powder possesses properties similar to those of cement and can, to some extent, replace it. However, its cementitious activity needs to be improved for optimal concrete applications [14]. This limitation can be mitigated by combining AOD slag with supplementary cementitious materials like fly ash, which enhances its reactivity through synergistic effects. However, the specific hydration mechanism of AOD slag in combination with fly ash remains largely unexplored in recycled concrete for pavement applications. Concepts such as microstructural development and mechanical properties need to be assessed. Understanding these fundamental relationships is essential for optimizing mixed designs and ensuring the adequate strength performance required for pavement applications.

Some scholars [15,16,17] have studied the physical and mechanical properties of recycled aggregates and compared them with those of natural aggregates. While these studies provided comprehensive reviews of various recycled aggregates for highway pavement applications (brick, concrete, and plastic) and their effects on concrete properties, they did not specifically investigate the combined use of recycled aggregates with industrial by-products like AOD slag and fly ash. The integration of multiple recycled materials represents an important area for sustainable concrete development. Our study addresses this gap by examining the specific interactions between recycled aggregates and AOD slag–fly ash cementitious systems, with particular attention given to the mechanical performance required for pavement applications. The results show that the addition of recycled aggregates reduces the strength of concrete within a controllable range, while also effectively reducing the cost of concrete. Ye Jinghui et al. [18] processed waste concrete blocks and prepared recycled aggregates according to standard grades. These aggregates were then mixed with cement, water, and necessary natural aggregates to prepare recycled concrete. This technology utilizes resources from construction waste. Some scholars [19] have conducted experimental research showing that when recycled aggregates are used to replace natural aggregates in the preparation of concrete, the material strength experiences a downward trend with increasing substitution rates. However, through technical means such as single-admixture or multi-admixture of slag, the strength of recycled concrete can be significantly improved [20], resulting in effective concrete products. Sun Jiaying et al. [21] found that the road performance of recycled aggregate permeable concrete is satisfactory, and its main road indicators meet the technical requirements. However, due to the inherent defects of recycled aggregates, it can only be applied to areas with low- and medium-grade traffic [22]. Although the strength of recycled concrete may decline, it can be improved, and its cost is low, resulting in more advantages than disadvantages.

In summary, this study utilized discarded treated AOD slag and fly ash as a replacement for part of the cement and discarded crushed recycled aggregates as a replacement for natural crushed stones to prepare AOD slag–fly ash recycled concrete. Unlike previous studies that examined these materials separately or in different applications, our research uniquely combines these waste materials in a systematic investigation specifically targeting pavement concrete applications. This study has several specific objectives. First, we investigate the compressive and flexural strengths of the cementitious material system of AOD slag–fly ash cement at different substitution rates. Second, we reveal the mechanism of interaction between AOD slag and fly ash and determine the optimal dosage through microscopic analysis. Third, we explore the influence of different replacement rates of recycled aggregates on the mechanical properties of concrete. Through this comprehensive approach, we aim to improve the resource utilization of steel slag and construction waste, promoting synergistic recycling of industrial solid waste and construction waste. Ultimately, this research fills a gap in the study of stainless steel slag applications in the field and provides a valuable reference for sustainable concrete pavement construction.

## 2. Materials and Methods

### 2.1. Materials

#### 2.1.1. Cementitious Materials

The cementitious material used in this test was Yufeng brand P.O 42.5 ordinary silicate concrete (Guangxi Yufeng Cement Co., Ltd., Liuzhou, China). The performance of the cement was tested in accordance with the requirements of the Chinese standard [23] (TJG3420-2020), and the results met these requirements [24,25] (GB175-2023, JTGTF30-2014). The main performance indicators are shown in Table 1.

Fly ash, the main type of solid waste discharged from coal-fired power plants, is often used as a substitute for cementitious material. Fly ash can fill tiny pores in concrete, enhance its density and impermeability, and reduce the amount of cement used, thus lowering costs. Its active ingredients can also react with other calcium compounds to form new hydration products, which enhance the strength and durability of the concrete. The main properties of the secondary fly ash used in this experiment are shown in Table 2.

**Figure 1:**
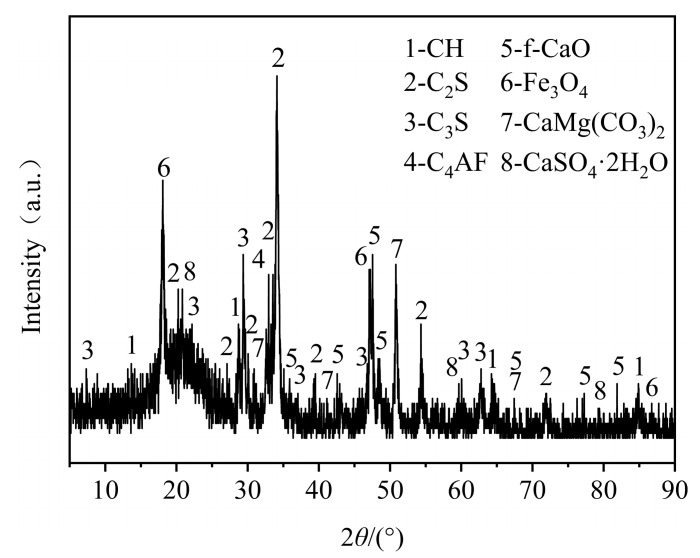
XRD pattern of AOD slag (Reprinted from Ref. [26]).

We used AOD slag produced by Liuzhou Iron and Steel (Group) Company in Guangxi (China). The AOD slag was crushed after treatment. To investigate the gelatinous substances present in the AOD slag, the crushed AOD slag was sieved through a 75 μm square-hole sieve to obtain AOD slag powder, which was grayish-white and loose in texture. Physical analysis of the AOD slag was conducted using X-ray diffraction (XRD) spectroscopy (Bruker AXS GmbH, Karlsruhe, Germany), and the results are shown in Figure 1. It was found that the AOD slag contained cementitious substances similar to cement, such as tricalcium silicate (CS_3_), dicalcium silicate (CS_2_), and tetrachalcium ferric aluminate (C_4_AF). Chemical composition analysis was carried out using X-ray fluorescence (XRF) spectroscopy, and the results are shown in Table 3. Unlike traditional slags (such as blast furnace slag or steel slag), AOD slag has a higher content of MgO and Cr_2_O_3_, and its hydration behavior is unique. Chemical components were identified similar to cement, including CaO, SiO_2_, MgO, MnO, TiO_2_, Cr_2_O_3_, and Fe_2_O_3_. This proved that AOD slag had cementitious activity.

#### 2.1.2. Aggregates

Machine-made sand (Yu’ang Building Materials Business Department Liuzhou, China) was used as the fine aggregate in the test, and the fineness modulus, measured according to JTGE42-2005 [27], was calculated using the following formula:

(1) $$M_{x}=\frac{\left(A_{0.15}+A_{0.3}+A_{0.6}+A_{1.18}+A_{2.36}\right)-5A_{4.75}}{100-A_{4.75}}$$

The test results are shown in Table 4. In the formula: M_x_—fineness modulus of sand. A_0.15_, A_0.3_, …, A_4.75_—are 0.15 mm, 0.3 mm, …, 4.75 mm cumulative residual percentage of each sieve.

**Figure 2:**
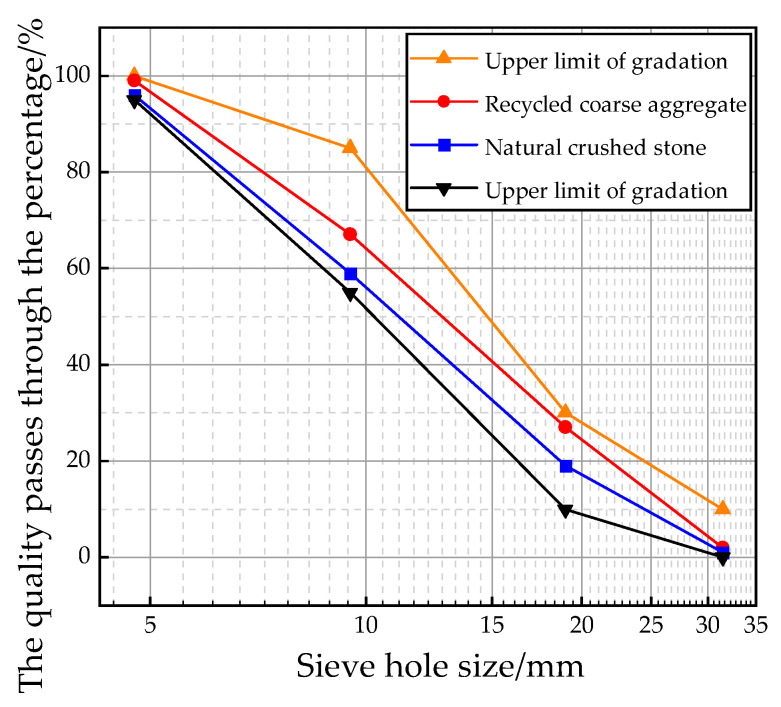
Coarse aggregate gradation curve.

The natural crushed stone used in the experiment was sourced from a quarry in Liuzhou. Natural crushed stone, formed by weathering and breaking rocks, contains a variety of particle sizes and good mechanical properties. Its irregular surface and edges allow for better bonding of cement while enhancing the strength of concrete. We passed natural crushed stone through square sieves of 4.75 mm and 31.5 mm, respectively. The recycled aggregate used in the test was discarded concrete obtained from a construction site in Liuzhou. Since the discarded recycled concrete was mostly demolished building components, it was crushed by a jaw crusher and then sieved to obtain particles ranging from 4.75 mm to 31.5 mm. The gradation of natural crushed stone and recycled aggregates is shown in Figure 2.

### 2.2. Sample Preparation and Mix Ratio

The mortar preparation test was conducted in accordance with the provisions of the Chinese standard “Test Method for Strength of Cement Mortar (ISO Method)” [28] (GB/T 17671-2021), and the admixtures were used to replace cement in equal mass. According to the “Technical Rules for Construction of Highway Cement Concrete Pavement” [25] (JTGTF30-2014), it is recommended that the admixtures do not exceed 30% of the cement. Therefore, the maximum substitution rate of a single admixture is 30%. The substitution rates of AOD slag were 0, 10%, 20%, and 30%, and those of fly ash were 0, 10%, 20%, and 30%. Orthogonal tests were established, and the mix ratios are shown in Table 5. The operation was carried out in accordance with the Chinese standard “Test Method for Strength of Cement Mortar (ISO Method)” (GB/T 17671-2021). After the mortar was mixed, it was formed using a vibration table. The samples were then demolded and placed into the standard curing box. Three samples were needed for each group, totaling 144 samples. The test ages of the samples were 3 days, 7 days, and 28 days.

Preparation of AOD–fly ash recycled concrete: Concrete specimens were prepared in accordance with the “Test Code for Cement and Cement Concrete in Highway Engineering of China” [23] (JTG3420-2020) to ensure a strength of C30 and a water–cement ratio of 0.47. The substitution rates of RA and AOD slag fly ash were used as variables, with RA substitution rates of 0, 30%, 50%, 70%, and 100%, respectively. The AOD slag–fly ash substitution rates were 0–30%, 10–20%, 20–10%, and 30–0%. The proportions of the concrete mix are shown in Table 6. The dimensions of the specimens to be cast are also provided. Each group contained various sizes of concrete, namely, 100 mm × 100 mm × 100 mm, 100 mm × 100 mm × 400 mm, and 150 mm × 150 mm × 300 mm, and three test blocks were poured, totaling 150 + 30 test blocks with a density of 2.35 g/cm^3^. After curing for 1–2 days at room temperature (20 °C ± 5 °C) and relative humidity exceeding 50%, samples were demolded and placed under standard conditions with a temperature of 20 °C ± 2 °C and relative humidity exceeding 95% for 28 days.

### 2.3. Methods

#### 2.3.1. Mortar Sample Strength Test Method

According to the Chinese standard [28] (GB/T 17671-2021), the STYE-300 microcomputer-controlled cement flexural and compressive strength-integrated machine (Zhejiang Tugong Instrument Manufacturing Co., Ltd., Hangzhou, China) was used to conduct three-point bending loading or flexural tests on cement mortar specimens. The specimens were uniformly loaded at a rate of 50 N/s ± 10 N/s until they broke, and the flexural strength data were recorded. After the flexural test, two broken specimens were taken out for the compressive test. They were loaded at a rate of 2400 N/s ± 200 N/s until the fracture specimen broke, and the compressive strength data was recorded.

#### 2.3.2. Microstructure Analysis

SEM tests were conducted on the damaged mortar samples using Hitachi Regulus8100 (Hitachi High-Tech Corporation, Tokyo, Japan). After sampling, some of the damaged gel samples were crushed into powder smaller than 0.075 mm, and XRD tests were conducted using the Brook D8 advance (Bruker AXS GmbH, Karlsruhe, Germany).

#### 2.3.3. Test Method for Concrete Specimens

Compressive strength, flexural tensile strength, axial compressive strength, and splitting tensile strength tests were conducted on concrete specimens using the RMT-301 multi-functional mechanical property testing machine (Institute of Rock and Soil Mechanics, Chinese Academy of Sciences, Wuhan, China) in accordance with Chinese code [23] (JTG3420-2020) The loading rates were 0.5 MPa/s, 0.05 MPa/s, 0.5 MPa/s, and 0.05 MPa/s, respectively.

The RC substitution rates in the table are 0%, 30%, 70%, and 100%. Since the changes in the substitution rates of F and AOD slag are consistent with those of the RC = 50% group, the mixed ratios of the other RC substitution rates were omitted.

## 3. Results

### 3.1. Orthogonal Test Results of Mortar Samples

**Figure 3:**
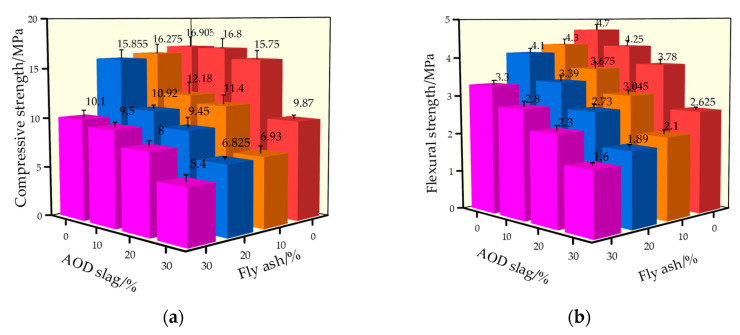

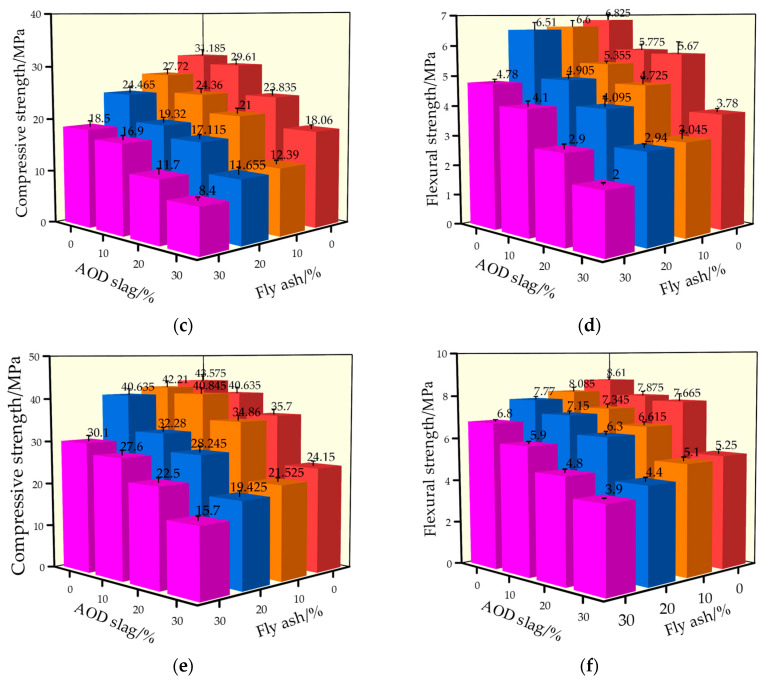
Sample strength diagram: (**a**) 3 days compressive strength; (**b**) 3 days flexural strength; (**c**) 7 days compressive strength; (**d**) 7 days flexural strength; (**e**) 28 days compressive strength; (**f**) 28 days flexural strength.

The flexural and compressive strengths of the mortar samples obtained through the above-mentioned mixed mortar designs are shown in Figure 3.

### 3.2. Mechanical Properties of AOD Slag–Fly Ash Recycled Coarse-Aggregate Concrete

#### 3.2.1. Compressive Strength

**Figure 4:**
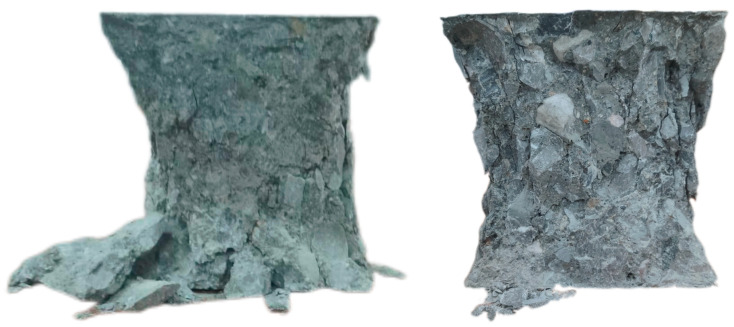
Forms of failure in compressive strength tests (150 mm × 150 mm × 150 mm).

**Figure 5:**
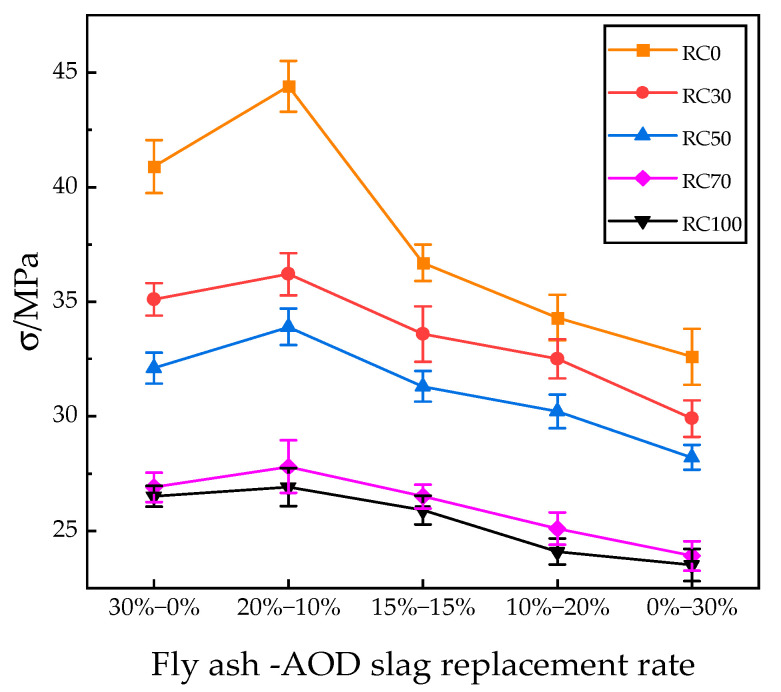
Compressive strength results of AOD slag–fly ash recycled concrete.

The compressive failure morphology of AOD slag–fly ash recycled concrete is similar to that of AOD slag–fly ash ordinary concrete: concave cylinders are eventually formed with larger ends and smaller middles, as shown in Figure 4. Compared to ordinary concrete, recycled concrete mainly experienced shear failure of the recycled aggregates themselves, as well as some bonding failures between the aggregates and the cementitious system materials. With the increase in the replacement rate of recycled aggregates, the brittle characteristics of the test blocks also significantly increase, leading to higher rates of failure. Figure 5 presents the compressive strength of concrete under the substitution rates of AOD slag and fly ash, both alone and in combination, as well as the substitution rate of recycled concrete.

#### 3.2.2. Flexural Tensile Strength

**Figure 6:**
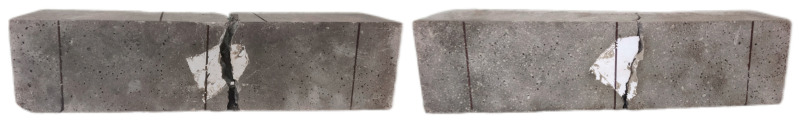
Forms of failure in flexural tensile strength test (100 mm × 100 mm × 400 mm).

**Figure 7:**
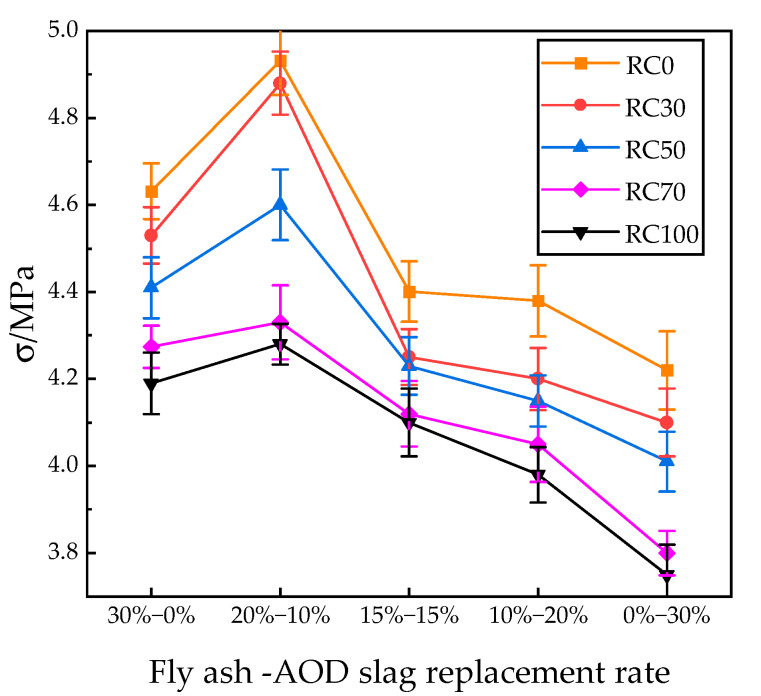
Effects of AOD slag, fly ash, and recycled aggregate substitution rates on flexural tensile strength.

When the flexural tensile strength test is conducted on a concrete specimen, the stress state is distributed in a triangular pattern, with the maximum tensile stress occurring toward the bottom. Since the compressive strength of the concrete specimen is much higher than the tensile strength, tensile cracking failure is initially shown at the specimen’s bottom, before the crack develops upward along the weak side of the specimen from the bottom and eventually breaks in the middle in the form of linear cracks. During the test, all flexural and tensile specimens broke from the middle, as shown in Figure 6. The results of the flexural tensile strength test are shown in Figure 7.

Based on the above results, when the proportion of recycled aggregates exceeds 50%, key mechanical performance indicators such as compressive strength and flexural tensile strength experience a significant downward trend in concrete samples. Considering the engineering application alongside the test data, the proportion of 50% was determined for recycled aggregates as the reference mix ratio in axial compression and splitting tensile tests.

#### 3.2.3. Axial Compressive Strength

**Figure 8:**
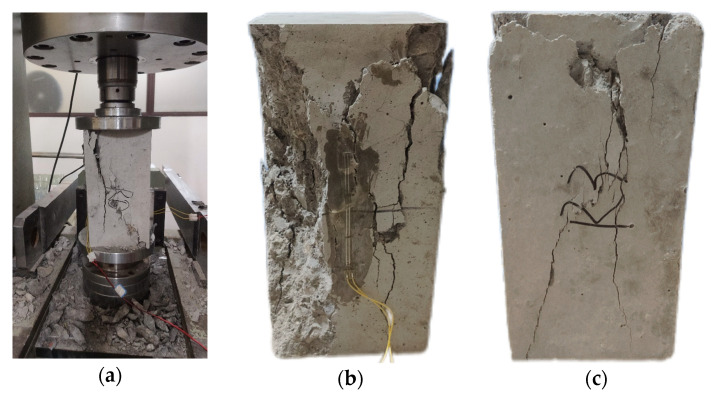

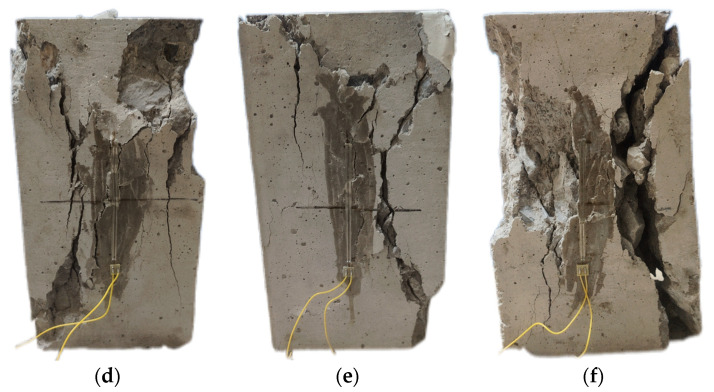
Forms of axial compressive failure in concrete: (**a**) axial compression test; (**b**) RC50F30A0; (**c**) RC50F20A10; (**d**) RC50F15A15; (**e**) RC50F10A20; (**f**) RC50F0A30 (150 mm × 150 mm × 300 mm).

**Figure 9:**
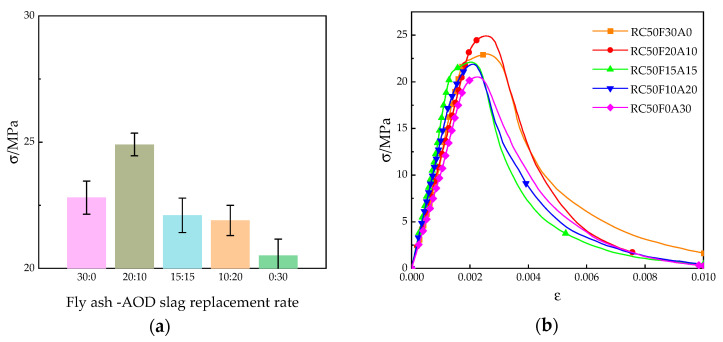
Results of axial compressive tests of concrete: (**a**) axial compressive strength of concrete; (**b**) axial compressive stress–strain curve of concrete.

After axial compression tests were performed on different mix ratios, no significant change was found in the failure patterns between different mix ratios, and the results did not differ significantly from ordinary concrete. As the pressure increased, the middle part of the specimens demonstrated signs of bulging, and fine hairline cracks (width < 0.1 mm) appeared on the surface, accompanied by intermittent cracking sounds. The micro-cracks extended into the mortar matrix, forming a local crack network. The main cracks penetrated the specimens, presenting a shear or longitudinal splitting failure pattern, with severe swelling in the middle; this led the aggregates and mortar to separate and detach, with a sudden drop in residual strength. The cracks were interconnected: aggregates broke away and began to detach from the specimen, as shown in Figure 8. Figure 9 shows the results of the axial compressive strength test of concrete.

#### 3.2.4. Splitting Tensile Strength

**Figure 10:**
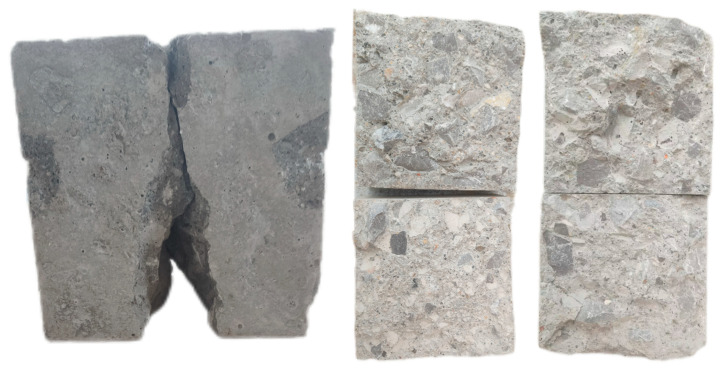
Forms of failure in cube splitting tensile strength test (150 mm × 150 mm × 150 mm).

During the splitting test, small cracks appeared on the side of the specimen as both the load and tensile stress in the middle of the specimen increased; eventually, the specimen split in half with a loud bang. Figure 10 shows that the recycled aggregates in AOD slag–fly ash recycled concrete mostly experienced fracture failures, while natural aggregates experienced bonding failures between cementitious materials. The results are shown in Table 7.

## 4. Discussion

### 4.1. Analysis of Orthogonal Test Results of Mortar Samples

#### 4.1.1. Effects of a Single Admixture on the Mechanical Properties of the Mortar Sample

1. The Influence of Fly Ash on the Mechanical Properties of the Mortar Samp

The strength changes in the mortar samples with fly ash substitution rates of 10%, 20%, and 30% are shown in Figure 5. As the flexural strength of the fly ash specimens gradually decreased compared to the control group (AF-00), their 28-day compression-flexural strength decreased by 3.3%, 7.2%, 44.7%, 6.4%, 10.8%, and 26.6%, respectively. When no more than 20% was added, fly ash had less effect on the compressive strength of the specimens.

This is because the chemical composition of fly ash is dominated by SiO_2_, which makes the surface of fly ash smooth and reduces the frictional force between particles. When fly ash is added, it is equivalent to diluting the cementitious material. Dilution is most pronounced when the fly ash content is 30%, at which point the compressive and flexural strength of the sample decreases the most.

2. The Effect of AOD Slag on the Mechanical Properties of Mortar Specimens

As shown in Figure 5, the samples with AOD slag exhibited a gradual increase in strength on days 3, 7, and 28; their strength was lower than that of the mortar specimens without any admixture. When the incorporation rate exceeded 20%, the decline in compressive–flexural strength became more pronounced. When the AOD slag content reached 30%, the compressive strength ratio, or activity index, was 81% and 73% on days 7 and 28, respectively, compared to mortar specimens without AOD slag. These values are higher than the current standard requirements of GB/T 20491-2017 [29], which specify 55% and 65%, and the strength growth pattern is consistent with the mortar without any admixture over time. Therefore, when the AOD slag content is ≤30%, the use of AOD slag as a cementitious material is reasonable.

The strength of fly ash samples was higher than that of AOD slag samples with the same dosage. When the dosage was 30%, the 28-day compressive and flexural strength of the fly ash sample was 29.5%, which is 24.6% higher than that of the AOD slag sample, respectively. This indicates that the content of cementitious substances in AOD slag is relatively low when used alone. Therefore, the admixture should be compounded.

#### 4.1.2. Effects of Compounding Admixtures on the Mechanical Properties of Cementitious Sand Samples

**Figure 11:**
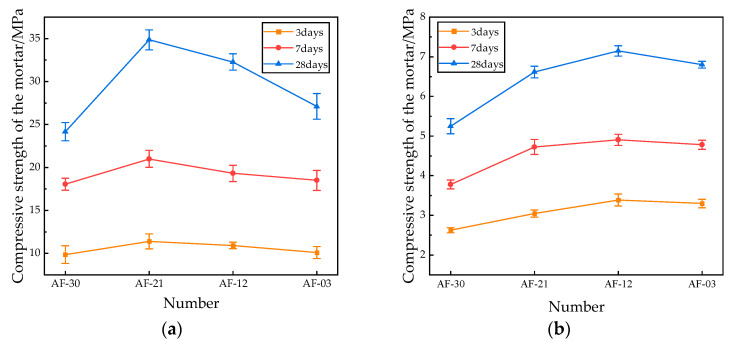
Mechanical properties of the mortar sample: (**a**) Compressive strength; (**b**) Flexural strength.

When the total admixture content was determined to be 30%, different admixture combinations were designed, as shown in Table 8, and the test results were obtained, as shown in Figure 11.

It can be seen from Figure 6 that when the admixture is AOD slag, the compressive and flexural strength of the sample is the lowest. With the addition of fly ash, the strength of the sample shows a significantly increasing trend before decreasing. When the ratio of AOD slag to fly ash was 20%:10%, the 28-day compressive strength of the specimens was optimal, with increases of 44.3% and 28.6%, respectively, compared to the specimens mixed with AOD slag or fly ash alone. When the ratio of AOD slag to fly ash was 10%:20%, the 28-day flexural strength of the specimens was optimal, with increases of 40% and 5.1% compared to the specimens mixed with either AOD slag or fly ash alone.

The main factors affecting the strength of the admixture sample were as follows: First, the average particle sizes of the fly ash and steel slag used were 24.77 μm and 32.42 μm, respectively; and the different particle sizes complemented each other, thus reducing the number of capillary pores in the slurry, making the sample denser and increasing the strength. Second, the alkaline environment provided by fly ash undergoes a secondary hydration reaction with AOD slag, generating more cementitious substances.

### 4.2. Microstructural Analysis and Mechanism Discussion of the Binder System

#### 4.2.1. XRD Analysis of Cementitious Sand

**Figure 12:**
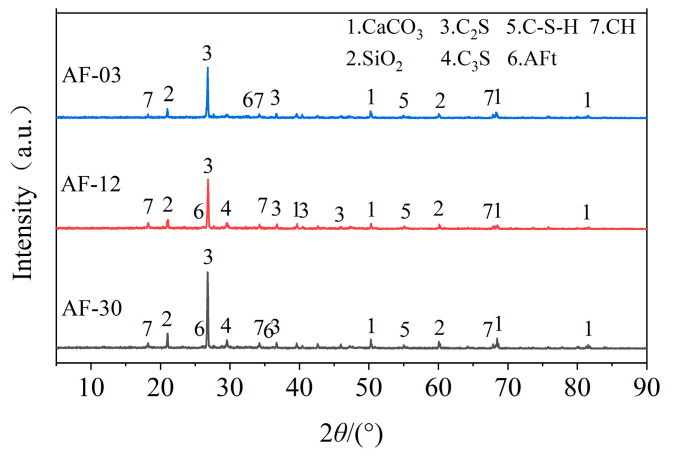
XRD pattern of the mortar sample after 28 days of curing.

To further analyze the hydration products of the AOD slag–fly ash cement system in different mixtures, X-ray diffraction (XRD) tests were conducted on three groups of mortar specimens (AF-03, AF-30, and AF-12) after 28 days of curing, as shown in Figure 12. All three samples contained the original mineral phases of AOD slag and cement, such as C_2_S and C_3_S, as well as the main hydration products of calcium hydroxide (CH), calcium silicate hydrate (C-S-H) gel, and ettringite (AFt). However, their relative contents varied among the samples.

When the replacement level was 10% AOD slag and 20% fly ash (AF-12), the peaks of SiO_2_ and C_2_S were significantly reduced, while those of C-S-H and AFt were markedly enhanced, indicating a more complete hydration process. In contrast, at lower or higher replacement levels (AF-03 and AF-30), stronger residual SiO_2_ and C_2_S peaks were observed, suggesting incomplete hydration.

Quantitatively, the variation in the intensities of C-S-H, CH, and AFt peaks corresponded well with the compressive-strength results of the mortars. The stronger C-S-H peak in AF-12 indicates a higher content of gel-like hydration products, which is consistent with its maximum flexural strength. These results demonstrate that an appropriate combination of AOD slag and fly ash promotes hydration reactions, forming a more stable and compact hydration structure that provides a microstructural basis for strength improvement.

#### 4.2.2. SEM Morphology Analysis of the Mortar

**Figure 13:**
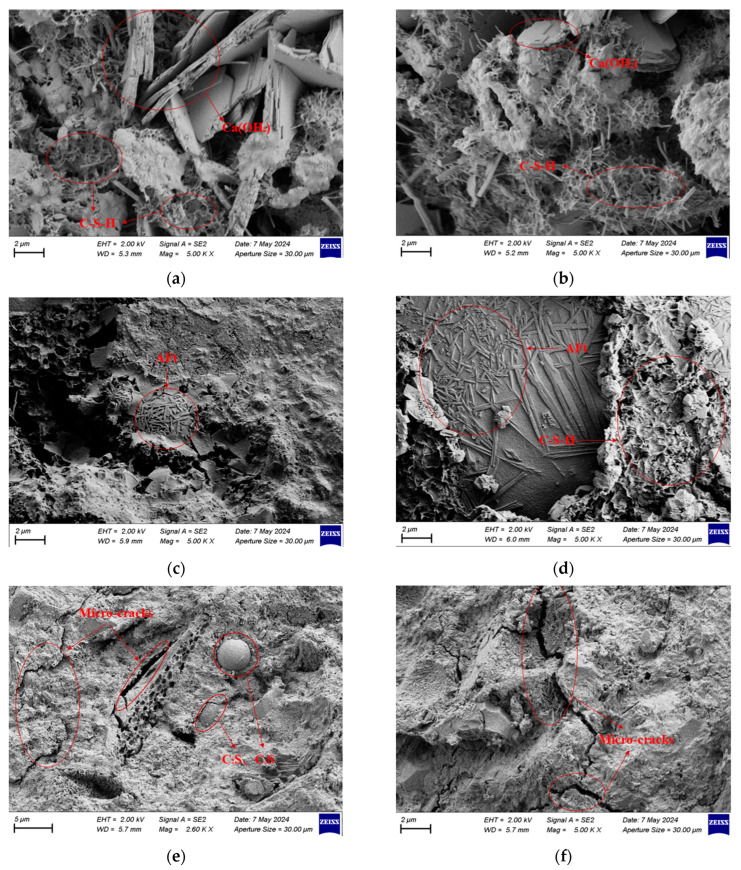
SEM image of the mortar sample magnified 5000 times after 28 days of curing: (**a**) Sample AF-03; (**b**) Sample AF-03; (**c**) Sample AF-12; (**d**) Sample AF-12; (**e**) Sample AF-30; (**f**) Sample AF-30.

Based on the XRD results, scanning electron microscopy (SEM) observations were carried out on the three groups of mortar specimens (AF-03, AF-12, and AF-30) after 28 days of curing, as shown in Figure 13.

As shown in Figure 13a,b, the AF-03 specimen contained CH crystals, flocculent C–S–H gel, and a small amount of needle-like AFt formed from cement hydration. The bonding between particles was loose, and noticeable pores were present. By contrast, the AF-12 specimen (Figure 13c,d) exhibited a large amount of flocculent C–S–H gel and needle-like AFt crystals filling the pores, resulting in the densest microstructure among all the samples. The AF-30 specimen (Figure 13e,f) exhibited a higher number of unhydrated spherical C_2_S and C_3_S particles, a lower amount of C-S-H formation, and reappearing pores, leading to a more porous and discontinuous structure.

A comparison between the microstructural features and the mechanical results indicates that the dense and continuous structure of AF-12 significantly improved the overall strength of the mortar. In contrast, AF-30 exhibited reduced structural continuity and lower strength due to the localized aggregation of hydration products and increased microcracking. The macroscopic mechanical behavior of the mortar corresponded well with the evolution of its microstructure.

#### 4.2.3. Mechanism Discussion

Based on the XRD and SEM analyses, the synergistic hydration between AOD slag and fly ash in the mortar system was identified as the main reason for the improvement in mechanical performance. During hydration, AOD slag releases calcium hydroxide (CH), which provides an alkaline environment that activates the reactive SiO_2_ in fly ash to undergo a secondary pozzolanic reaction, forming additional calcium silicate hydrate (C-S-H) gel. This secondary reaction accelerates the hydration process of the binder, leading to a denser matrix structure, reduced porosity, and enhanced strength. At the optimal replacement level (10% AOD slag + 20% fly ash), the weakened SiO_2_ peaks and strengthened C-S-H peaks in the XRD patterns, together with the reduced porosity observed in SEM images, confirm the existence of this synergistic effect.

Notably, this mechanistic analysis is based on the microstructural results of the mortar system, reflecting the intrinsic reactivity of the binder materials. Similar strength trends observed in the recycled concrete tests (Section 4.3) suggest that the same mechanism may also operate in the concrete system; however, the microstructural characteristics of the aggregate–paste interface were not characterized in this study and should be investigated in future research.

### 4.3. Mechanical Property Analysis of AOD Slag–Fly Ash Recycled Coarse Aggregate Concrete

#### 4.3.1. Analysis Compressive Strength

It can be seen from Figure 5 that the compressive strength of concrete decreases significantly as the rate of replacement between natural aggregates and recycled aggregates increases. When the replacement rates were 30%, 50%, 70%, and 100%, the compressive strength decreased by up to 18.47%, 23.64%, 37.39%, and 39.41%, respectively, compared to the control group (RA = 0).

The main reason for this is that the higher impurity content and the old mortar attached to the surface of recycled aggregates damage the overall integrity of the concrete and hinder bonding with the new cement paste, thereby forming a weak interfacial transition zone (ITZ) and reducing the compressive strength [30].

As the replacement ratio of fly ash decreases from 30% to 0% and that of AOD slag increases from 0% to 30%, the compressive strength of concrete shows a “first increase and then decrease” trend. When the combined replacement of fly ash and AOD slag is 20–10%, the compressive strength reaches its peak, which is 8.57% and 36.2% higher than that of fly ash and AOD slag alone. This indicates that the blended system exhibits superior performance compared with single-admixture systems. When the replacement ratios of recycled aggregates are 50% and 70%, the compressive strengths are 33.9 MPa and 27.8 MPa, respectively. Compared with previous studies on recycled concrete with similar replacement ratios (approximately 30 MPa) [31], the compressive strength decreased by only 7.3% when AOD slag and fly ash were used as partial cement replacements. Meanwhile, cement consumption was reduced by 30%, and the compressive strength still met the C30 concrete strength requirement, indicating that this replacement ratio is feasible.

The improvement in strength at this replacement level can be attributed to two main reasons. Firstly, the smaller and differently sized particles of fly ash and AOD slag can complement each other to fill the voids between aggregates, thereby improving the compactness of the concrete and enhancing its strength. Secondly, as revealed by microstructural analysis, there is a significant synergistic effect between AOD slag and fly ash. During hydration, calcium hydroxide (CH) released from AOD slag provides an alkaline environment that activates the reactive SiO_2_ in fly ash to undergo secondary hydration, producing additional calcium silicate hydrate (C-S-H) gel. This process improves the interfacial transition zone (ITZ) of recycled concrete and enhances its overall strength [5].

#### 4.3.2. Analysis of Flexural Tensile Strength

The curves of the flexural tensile strength test results are shown in Figure 7. The variation pattern is basically the same as that of the compressive strength. When the replacement rates of recycled aggregates are 30%, 50%, 70%, and 100%, the maximum decreases in flexural tensile strength are 4.11%, 6.73%, 12.21%, and 13.22%, respectively. The curves showed a trend of first increasing and then decreasing with the change in the fly ash-AOD slag substitution rate. The optimal substitution rate was still 20–10%, which increased the flexural tensile strength by 6.08% and 14.44%, respectively, compared to concrete using fly ash or AOD slag only, further verifying the accuracy of the test results.

#### 4.3.3. Analysis of Axial Compressive Strength

As shown in Figure 9, the test data indicate that the compounding ratio of fly ash and AOD slag has a significant regulatory effect on the axial compressive strength of concrete. When the content of fly ash–AOD slag is 20:10, the axial compressive strength of the concrete reaches a peak of 24.9 MPa, which is 9.2% and 21.5% higher than that of pure fly ash and pure AOD slag, respectively. This phenomenon is due to the filling effect of micro-aggregates and the synergistic effect of active components in AOD slag (such as CaO, Al_2_O_3_). Fly ash and AOD slag refine the pore structure [32,33], reducing the internal voids of concrete. Meanwhile, AOD slag generates more C-S-H gel through secondary hydration reaction, enhancing the compactness of the matrix. However, when the AOD slag exceeds 10%, the strength shows a decreasing trend, indicating that excessive amounts of AOD slag lead to a certain degree of failure for the synergistic effect with fly ash. The total amount and generation rate of hydration products in the entire system decrease. The interface bonding force between the unhydrated AOD slag particles and the cement paste is weak and becomes the origin for micro-cracks, which reduces the axial compressive strength of the concrete. This phenomenon is consistent with the observation results of Roslan et al. [34] when studying the steel slag–fly ash composite system. Despite 10% steel slag being the optimal dosage, they found that an excessively high dosage will affect the early development of strength, especially under short-term curing conditions.

By comparing the stress–strain curves of five groups of concrete, when AOD slag was added, the decline in the axial compressive strength of the concrete became steeper and then slower. This indicates that when AOD slag exceeds 10%, the brittleness of the concrete begins to change. This is mainly because AOD slag undergoes a secondary hydration reaction with fly ash, generating cementitious substances that fill the micro-cracks in the concrete, resulting in denser concrete. As a result, the concrete samples mixed with 10% AOD slag exhibit the most brittle characteristics, while the remaining groups have incomplete or no secondary hydration reaction, and the internal structure of the samples is relatively loose. Therefore, the brittleness of the samples was 10% lower than that of the AOD slag group. This is consistent with the research findings of Qiang et al. [35], who pointed out that under appropriate water–cement ratio conditions and the optimal steel slag dosage can enhance the stress–strain behavior of cement-based materials, but an excessively high dosage (especially under high water–cement ratio conditions) will have an adverse effect on the mechanical properties of concrete.

#### 4.3.4. Analysis of Splitting Tensile Strength

This test systematically investigated the influence of the composition of cementitious materials on the splitting tensile strength of concrete by adjusting the blending ratio of fly ash and AOD slag. The results are shown in Table 8. When the ratio of fly ash to AOD slag was 20:10, the split tensile strength of the concrete reached a peak of 3.4 MPa, which was 3.0% higher than that of the pure fly ash group and 61.9% higher than that of the pure AOD slag group, respectively. The splitting tensile strength was close to 3.6 MPa of recycled concrete with the same proportion and admixture [36]. The micro-aggregate filling effect of fly ash and the secondary hydration reaction of active components in AOD slag worked together to reduce matrix porosity and enhance the interfacial bond between aggregates and matrix, thereby improving tensile performance. When the AOD slag content exceeded 10%, the splitting strength decreased to 3.0 MPa and 2.8 MPa, respectively. This is attributed to the unreacted particles and pore defects introduced by excess AOD slag, which led to stress concentration and rapid crack propagation.

## 5. Conclusions

This study investigated the preparation of pavement concrete in which AOD slag and fly ash were used to partially replace cement as the supplementary cementitious material. Recycled aggregate was used to partially replace natural coarse aggregate. The mechanical properties and microstructural characteristics of mortar specimens were analyzed to reveal the hydration mechanism of the blended binder system and its influence on strength development, while the mechanical performance of AOD slag–fly ash recycled concrete was further evaluated.

(1) In the mortar system, the incorporation of AOD slag and fly ash significantly improved the hydration activity and strength compared with single-admixture systems. The flexural strength exhibited a “first increase and then decrease” trend with increasing replacement levels, which reached their peak when the mixture contained 10% AOD slag and 20% fly ash.

(2) According to the microstructural observations and mechanistic interpretation of the AOD slag–fly ash–cement system, a synergistic effect was identified between AOD slag and fly ash. The hydration of AOD slag releases calcium hydroxide (CH), providing an alkaline environment that activates the reactive SiO_2_ in fly ash to undergo a secondary hydration (pozzolanic) reaction, generating additional calcium silicate hydrate (C-S-H) gel. This synergy leads to higher strength in the blended system compared to when either material is used alone.

(3) In the recycled concrete system, when the replacement ratio of recycled aggregate was 50% and the admixture ratio are the same (10% AOD slag + 20% fly ash), all four mechanical strength indices reach their optimum values: compressive strength at 33.9 MPa, flexural strength at 4.6 MPa, axial compressive strength at 24.9 MPa, and splitting tensile strength at 3.4 MPa. All four indices followed a “rise-then-fall” trend consistent with that observed in the mortar system.

(4) It should be emphasized that the identified “optimal mixture” (10% AOD slag, 20% fly ash, and 50% recycled aggregate) represents an empirical observation under the specific experimental conditions of this study (w/b = 0.47; curing temperature 20 ± 2 °C; relative humidity > 95%; and 28 days of standard curing), rather than a universal guide. Different water–binder ratios, curing regimes, chemical compositions, and recycled aggregate qualities may produce variations in the optimal replacement range.

Overall, the recycled concrete system incorporating 10% AOD slag and 20% fly ash with 50% recycled aggregate not only maintains or improves mechanical performance but also significantly reduces cement consumption and CO_2_ emissions, resulting in both economic and environmental benefits. This composite system is suitable for use as pavement bases or other non-structural concrete applications that require sustainable use. Before engineering applications, site-specific mix trials and durability evaluations (including freeze–thaw cycles, wet–dry alternation, and chloride and sulfate attacks) are recommended. Future studies should combine TG-DTG and MIP analyses to quantitatively investigate the evolution of hydration products, assess the long-term chemical stability of AOD slag, and explore the multi-scale mechanisms affecting material durability.

## Figures and Tables

**Table 1:** Basic performance indicators of cement.

Specific Surface Area	Setting (min)		Compressive Strength/Mpa		Flexural Strength/Mpa	
/	Initial setting	Final coagulation	3 days	28 days	3 days	28 days
(m^2^/kg)						
365	188	235	17.1	43.6	4.7	8.6

**Table 2:** Main properties of fly ash.

Test Indicators	Test Method	Specification Requirements	Results of Use
SiO_2_	T0816	>70	70.2
Loss on ignition (%)	T0817	≤20	2.8
Specific surface area (cm^2^/g)	T0820	>2500	3162
0.3 mm sieve pass rate (%)	T0818	≥90	100
0.075 mm sieve pass rate (%)	T0818	≥70	89.6
Moisture content (%)	T0801	≤35	0.85

**Table 3:** Chemical composition of AOD slag (Reprinted from Ref. [26]).

CaO/%	SiO_2_/%	Fe_2_O_3_/%	MgO/%	TiO_2_/%	Cr_2_O_3_/%	MnO/%	K_2_O/%	SO_3_/%
47.38	26.20	13.80	5.29	3.39	2.31	1.22	0.18	0.11

**Table 4:** Machine-made sand sieve residue percentage/%.

**Manufactured Sand**	**Cumulative Sieve Residue Percentage/%**					
	0.15	0.3	0.6	1.18	2.36	4.75
	94.5	79.3	59.2	34.5	19.5	7.5

The sand ratio was calculated to be 2.70 throughout the experiments.

**Table 5:** Mortar test mix ratio design.

Number	Water (g)	ISO Sand(g)	Cementitious Materials		
			Cement/%	AOD Slag/%	Fly Ash/%
AF-00	225	1350	100	0	0
AF-10			90	10	0
AF-20			80	20	0
AF-30			70	30	0
AF-01			90	0	10
AF-11			80	10	10
AF-21			70	20	10
AF-31			60	30	10
AF-02			80	0	20
AF-12			70	10	20
AF-22			60	20	20
AF-32			50	30	20
AF-03			70	0	30
AF-13			60	10	30
AF-23			50	20	30
AF-33			40	30	30

**Table 6:** Design of mixed concrete test proportions.

Number	Recycled Aggregates/kg	Natural Aggregates/kg	Cement/kg	Water/kg	Manufactured Sand/kg	Fly Ash/kg	AOD Slag/kg
RC50F30A0	676.3	676.3	116.9	112.1	696.8	71.6	0
RC50F20A10						47.7	23.9
RC50F15A15						35.8	35.8
RC50F10A20						23.9	47.7
RC50F0A30						0	71.6

Note: RC is the replacement rate of the recycled aggregate, in %; F is fly ash; A is AOD slag; RC50F20A10 is concrete with a 50% regenerated aggregate replacement rate, 20% fly ash content, and 10% AOD slag content.

**Table 7:** The splitting tensile strength of AOD slag–fly ash recycled concrete.

Number	RC50F30A0	RC50F20A10	RC50F15A15	RC50F10A20	RC50F0A30
Split tensile strength /MPa	3.3	3.4	3.0	2.8	2.1
Standard deviation	0.125	0.086	0.092	0.129	0.072

**Table 8:** Mix ratio of 30% admixture.

Number	Cementitious Materials		
	Cement/%	AOD Slag/%	Fly Ash/%
AF-30	70	30	0
AF-21		20	10
AF-12		10	20
AF-03		0	30

## Data Availability

The original contributions presented in this study are included in the article. Further inquiries can be directed to the corresponding author.
